# Modernizing U.S. Sunscreen Regulations: How Newer Filters Can Improve Public Health

**DOI:** 10.1111/phpp.70032

**Published:** 2025-08-08

**Authors:** Clayton Wade Turner, Leslie Torgerson

**Affiliations:** ^1^ Rocky Vista University School of Osteopathic Medicine Parker Colorado USA

**Keywords:** FDA regulation, photoprotection, public health, skin cancer prevention, sunscreen, UV filters

## Abstract

**Background:**

Skin cancer is the most common cancer in the United States, with over 5 million cases diagnosed annually. Despite being largely preventable, incidence rates continue to rise, largely due to inadequate protection from ultraviolet (UV) radiation, the primary environmental cause of skin cancer. Although global innovation has advanced sunscreen technology, the U.S. regulatory system has not kept pace, limiting public access to more effective UV filters.

**Methods:**

This review examines the current U.S. Food and Drug Administration (FDA) sunscreen filter regulatory framework, including the Generally Recognized As Safe and Effective (GRASE) criteria and maximal usage trial (MUsT) protocols. We compare U.S. approved filters with newer filters used internationally and analyze barriers to their domestic approval.

**Results:**

International markets currently use advanced filters such as bemotrizinol, bisoctrizole, drometrizole trisiloxane (DT), and terephthalylidene dicamphor sulfonic acid (TDSA), which offer broader and more stable UV protection. These filters have demonstrated favorable safety and efficacy profiles but remain unavailable in the U.S. due to regulatory inertia. The FDA's new Over‐the‐Counter (OTC) Monograph Order Request (OMOR) process may provide a pathway to modernize sunscreen regulations.

**Conclusion:**

Modernizing U.S. sunscreen regulations to allow approval of newer, evidence‐backed UV filters is a critical step in improving public health. Broader access to more effective sunscreens could play a significant role in reducing the incidence of UV‐induced skin cancer.

## 1 Introduction

Melanomas of the skin are considered the fifth most common type of cancer in the U.S. [1] The prevalence of skin cancer in the U.S. is substantial, with more than 5 million cases being treated annually, with an estimated cost of over $8 billion [2]. In 2022 alone, skin cancers accounted for 8243 deaths [3]. In addition, the annual incidence rate of skin cancers continues to increase; in fact, the rates have more than tripled since 1975, from 8.8 new cases per 100,000 people to 27.3 new cases per 100,000 people in 2021 [1].

Sunscreen plays an important role in preventing damage caused by the sun's harmful UV rays [4]. In fact, daily use of sunscreen reduces the risk of developing melanoma by half [4]. UVB and UVA are two types of radiation that can cause damaging effects on health. UVA has a longer wavelength range from 315 to 400 nm, whereas UVB has a shorter wavelength range from 280 to 315 nm. UVA comprises about 95% of terrestrial radiation, whereas UVB is about 5% [5]. UVB is associated with skin burning because it penetrates and damages the outermost layers of the skin. On sunscreen labels, the SPF number is connected to UVB radiation. UVA, on the other hand, is associated with skin aging because it penetrates the skin deeper than UVB rays, causing genetic damage to cells in the innermost portion of the epidermis. On sunscreen labels, the term “broad‐spectrum” indicates that the product contains protection against UVB as well as UVA radiation [6].

## 2 Overview of Sunscreen Regulations in the U.S. Versus Europe and Asia

In the U.S., sunscreen ingredients such as UV filters are regulated as over the counter (OTC) drugs, meaning they must undergo rigorous testing for safety and efficacy before receiving FDA approval. This process can take years, and the FDA has not approved any new sunscreen filters since the 1990s, despite the availability of more advanced filters in other countries [7].

In contrast, countries like South Korea and those in the European Union treat sunscreens as cosmetic products, allowing for a more streamlined approval process [8, 9]. As a result, consumers in these regions have access to a broader range of UV filters that are often more advanced in terms of formulation and cosmetic appeal. The European Union and Korea both use filters that provide superior protection against UVA/UVB radiation compared to the U.S. [10].

One advantage of updating sunscreen filters to those used in other countries includes a decrease in skin cancer rates. Also, more effective sunscreens could help decrease photoaging, reducing the prevalence of wrinkles, pigmentation, and other sun‐induced skin damage [9, 11].

However, newer sunscreen filters, though more effective, still carry the risk of allergic reactions or irritant dermatitis in certain individuals [12]. However, these adverse events will have been investigated and tracked prior to approval through human repeat insult patch tests, allergenicity, sensitization, and phototoxicity testing [13]. In addition, adverse events are recorded and stored in databases in countries that regulate sunscreens as drugs [14, 15]. In Australia, for example, the Therapeutic Goods Administration (TGA) tracks all adverse events to drugs and their formulations through the Database of Adverse Event Notifications (DAEN) system [15].

Given these gaps in sunscreen regulation, this review hypothesizes that incorporating internationally recognized sunscreen filters into U.S. regulations would enhance UV protection and reduce skin cancer rates. This paper explores these regulatory differences and advocates for adopting international best practices to improve sunscreen availability and effectiveness in the U.S., ultimately enhancing public health and reducing the incidence of skin cancer. By examining the regulatory frameworks in Europe and Asia, evaluating the efficacy and safety of advanced UV filters, and addressing the challenges within the U.S. regulatory system, we can develop actionable recommendations to modernize U.S. sunscreen regulations.

## 3 US Filters

Approval of sunscreens in the US goes through the FDA OTC monograph, which categorizes OTC medications. There are three categories for these products: category I is GRASE, category II is not GRASE, and category III includes products for which more data is needed [16]. In the US, there are currently 16 UV filters approved for use by the FDA [7]. (accessed 8/12/2024). These filters are listed in Table 1, along with their maximum concentration percentages. Of these, only two, zinc oxide and titanium dioxide, are recognized as category I. The rest are considered category III [7]. Although 16 filters are available for use, not all are commonly used by the industry, limiting the availability of modern sunscreens that meet the needs of diverse skin types [17]. The list of approved filters has not been updated since 1999. In September 2021, however, the FDA proposed an update to the requirements for sunscreens through the Coronavirus Aid, Relief, and Economic Security (CARES) Act [18]. Although this proposal does not include new approved filters, it does include five new updates to the over the counter (OTC) drug monograph requirements for sunscreens:

1. Zinc oxide and titanium dioxide are proposed to be generally recognized as safe and effective (GRASE) for sunscreen use, whereas PABA and trolamine salicylate are proposed as not safe and effective for sunscreen use. The remaining 12 filters need more safety information [13].
2. Sprays, oils, lotions, creams, gels, butters, pastes, ointments, and sticks are proposed as safe and effective. More data is needed for powders.
3. Raise the maximum proposed labeled SPF from SPF 50+ to SPF 60+, require any sunscreen SPF 15 or higher to be broad spectrum, and require for all broad‐spectrum products SPF 15 and above, as SPF increases, broad spectrum protection increases.
4. Include alphabetical listing of active ingredients on front panel. Require sunscreens SPF 15 and below to include “See Skin Cancer/Skin Aging alert” on the front panel. Require font and placement changes to ensure SPF, broad spectrum, and water resistance statements stand out.
5. Sunscreen‐insect repellent combination products proposed not safe and effective [19].

**TABLE 1:** Sunscreens approved for use in the US and their maximal concentrations.

Filter	Maximal concentration (%)
Aminobenzoic acid (PABA)	15
Avobenzone	3
Cinoxate	3
Dioxybenzone	3
Ensulizole	4
Homosalate	15
Meradimate	5
Octinoxate	7.5
Octisalte	5
Octocrylene	10
Oxybenzone	6
Padimate O	8
Sulisobenzone	10
Titanium dioxide	25
Trolamine salicylate	12
Zinc oxide	25

The FDA approves sunscreens through two different routes: the OTC drug monograph process mentioned previously, and the new drug application (NDA) process [20]. New sunscreens may be approved under the NDA or the abbreviated NDA. Sunscreens that were not in use before the inception of the OTC drug review are not eligible for approval through the OTC drug monograph process [20]. The OTC monograph is a list of guidelines that establishes conditions that allow drugs to be considered generally recognized as safe and effective for OTC use (GRASE). Drugs that are considered GRASE do not need to go through the NDA process [13].

In the OTC monograph, the only two filters that are currently considered GRASE are zinc oxide and titanium dioxide. These two sunscreens are physical filters, whereas the rest of the filters on the list are considered chemical. The reason for this change in the monograph is the possibility of adverse side effects with chemical sunscreens. Two chemical‐based filters on this list are especially concerning due to their potential health risks. Oxybenzone and homosalate have been evaluated by the European Commission Scientific Committee on Consumer Safety (SCCS) in 2021 as having potential endocrine disrupting properties. Because of these properties, they recommend maximal concentrations of 2.2% and 0.5% for oxybenzone and homosalate, respectively [21, 22]. That contrasts with the US maximal limits for oxybenzone and homosalate of 6% and 15%, respectively.

The conditions listed in the OTC monograph for sunscreens are consistent with the requirements for chronic‐use topical drug products. Chronic use is defined by the FDA as regular, prolonged use over an extended amount of time. Generally, using a product for longer than 6 months, either continuously or intermittently, would be considered chronic use [23]. These conditions include topical safety data, bioavailability, and evaluation of adverse events observed in clinical studies [7].

Two adverse events that are commonly observed in clinical studies include photoallergy and phototoxicity. These side effects are usually attributed to the sunscreen's formulation, including the UV filter used. Photoallergy is a type IV delayed hypersensitivity reaction that requires prior sensitization [24]. This process occurs when a chemical is activated by UV light into a reactive species that binds with dermal proteins. The classic clinical vignette of a photoallergy consists of wheal and flare response, dermatitis, edema, and vasodilation with onset occurring within 24–72 h of exposure [25]. Topical and/or systemic exposure of the offending agent may elicit a photoallergic response. Topical reactions are termed photocontact dermatitis, as opposed to systemic photoallergy [24]. Phototoxicity, on the other hand, differs from photoallergy in that the offending agent causes a direct response as opposed to a delayed hypersensitivity reaction seen in photoallergies [26].

When researchers formulate new sunscreens, they are required to follow a specific process. For example, human dermal sensitization, irritation, and photosafety studies are required to be conducted at the highest concentration at which a GRASE determination is sought, using appropriate vehicles alone and with positive and negative controls. Sensitization studies need to be conducted in three phases: induction, rest, and challenge phases. Irritation studies need to evaluate erythema, edema, and a papular response or skin erosion [7]. The human photosafety study consists of an assessment of photoallergies and phototoxicities [7]. A maximal use trial (MUsT) is conducted as part of the bioavailability and pharmacology assessment, and captures the effect of maximal use on absorption into the blood [7]. Systemic carcinogenic studies and developmental and reproductive toxicity studies are needed, unless MUsT resulted in a steady state blood level < 0.5 ng/mL [7].

A key factor to consider in the safety of UV filters is the rising use of sunscreens by the public. According to a poll conducted by Civic Science, which included 311,844 responses from May 29, 2015, to May 21, 2024, sunscreen usage has increased from 69% in 2015 to 75% in 2024 [27]. Another source indicates that in 2016 a total of 161,882,779 sunscreen units were sold in the United States, up from 153,870,186 in 2011 [28]. This represents a 5.21% increase in sunscreen sales over the course of 5 years. But are these sunscreens as effective as they are touted to be?

In 2021, a study was published that evaluated 51 sunscreens with SPF values from 15 to 110 that are sold in the US using the ISO 24443:2012 method to assess UVA protection. In vitro SPF values from lab‐measured UV absorption and computer modeling were on average 42% and 59% of the labeled SPF [29]. The same study showed that the majority of products assessed provided an average of 24% of the labeled UVA protection factor [29]. This study indicates the need for increased regulation of manufacturing processes to ensure sunscreen efficacy as well as the need for increased production and use of broad spectrum UV protection.

## 4 Comparison of Regulatory Processes

Countries in Europe and Asia classify sunscreens as cosmetics rather than drugs. This regulatory approach allows for a faster and more efficient approval process. For example, Europe's Cosmetic Products Regulation (CPR) facilitates the introduction of new UV filters through a less cumbersome safety assessment process compared to the FDA's requirements [30]. Similarly, South Korea's innovative cosmetic regulations have led to the rapid adoption of advanced filters previously mentioned.

The European Union's CPR requires that cosmetic products, including sunscreens, undergo a safety assessment conducted by a qualified person. This assessment includes a review of the product's ingredients, intended use, and potential risks to human health. Although this process is rigorous, it is also more flexible and adaptive than the FDA's drug approval process, enabling the timely introduction of new and improved UV filters.

In South Korea, skincare products are regulated by the Ministry of Food and Drug Safety (MFDS), with many products categorized as “quasi‐drugs,” allowing for a relatively streamlined approval process compared to pharmaceuticals [8]. This enables Korean companies to frequently release innovative products, catering to the demand for cutting edge beauty trends. These products are often supported by consumer testing and nonclinical studies.

In contrast, the US FDA requires a more rigorous approval process for skincare products, which necessitates extensive testing for safety, efficacy, and stability. Although the FDA has released a roadmap to implement more modern drug testing standards, it still requires preclinical animal testing and does not allow for more modern testing methods, such as in vitro, in silico, or mechanistic toxicological safety and risk assessment [31]. As a result, it can take longer for certain skincare products, especially those with newer UV filters or active ingredients, to gain approval for the US market.

An important factor to consider when evaluating the popularity of these newer sunscreens is the way they are marketed. One difference is that in the US, sunscreens are marketed using only the SPF scale, whereas in Korea and other countries, the Protection Grade of UVA (PA) system is used in addition to the SPF scale. The SPF scale measures protection against UVB rays, which cause sunburn and contribute to skin cancer. The higher the SPF rating, the longer the skin is protected from UVB exposure. In contrast, the PA system focuses on protection against UVA rays, which penetrate deeper into the skin and cause premature aging and contribute to skin cancer. The PA system uses a scale of plus signs (PA+, PA++, PA+++, PA++++), where more plus signs indicate stronger protection against UVA [32]. The EU uses a similar scale to assess UVA protection, called the persistent pigment darkening (PPD) protection factor, which can be converted to the PA system [32]. Adopting a UVA scale in the US could help clarify information for the consumer, allowing them to make better informed decisions regarding their health.

## 5 Review of New Filters

### 5.1 Bemotrizinol and Bisoctrizole

Bemotrizinol and Bisoctrizole are ultraviolet filters that are currently used in countries outside of the US. Bisoctrizole and Bemotrizinol are both considered organic broad spectrum UV filters, meaning they absorb UVA and UVB radiation [32]. Bemotrizinol has two UV absorption peaks, one at 310 nm and the other at 340 nm. Bisoctrizole, on the other had peaks around 360 nm, according to the manufacturer, BASF [33]. Bemotrizinol is currently being sponsored for approval in the U.S. by DSM‐Firmenich through the FDA's new Over‐the‐Counter (OTC) Tier 1 Monograph Order Request (OMOR) process. Potential GRASE determination expected in March of 2026. If approved, Bemotrizinol will be first new Category 1 UV filter added to OTC monograph that has been tested under FDA's GRASE and MUsT safety and efficacy standards [34, 35].

Bisoctrizole and Bemotrizinol have been approved in the EU for two main reasons: filter size and low level of photosensitivity [36]. The large structure and high molecular weight, compared to existing filters, of both bisoctrizole and bemotrizinol allow them to sit on top of the skin. An in vitro study on dermal penetration in rats and humans shows that bisoctrizole has minimal skin absorption, with approximately 0.01% of the applied dose penetrating human skin and 0.06% penetrating rat skin. Although dermal uptake is low, the possibility of bioaccumulation from repeated skin applications cannot be ruled out [37].

Studies have been performed to assess the possibility of endocrine disrupting effects on these filters [38, 39]. According to one study conducted on rats, it was found that both bisoctrizole or bemotrizinol did not provide a positive response to either androgen or estrogen receptor competitive binding assays [39].

A recent preliminary clinical pharmacokinetic evaluation of bemotrizinol found that concentrations rarely exceeded the FDA's limit of 0.5 ng/mL in plasma. They also found that there were few adverse effects, which were mostly considered mild [40]. This supports the opinion that 6% bemotrizinol is safe for human sunscreen use. It is worth noting that the maximum allowed concentration for sunscreen use is 10% for the EU, MERCOSUR, Australia, Korea, China, and ASEAN. For Canada, the maximum concentration is 6% [40]. The current OMOR for BEMT in the US is also supporting a maximum concentration of 6% [35].

For comparison, a different study published in 2019 compared the plasma concentrations of avobenzone, oxybenzone, octocrylene, and TDSA. In their study, it was found that the geometric mean maximum plasma concentrations > 0.5 ng/mL were reached for all four filters after four applications. Interestingly enough, the most common side effect was a mild rash that occurred in only one participant [41]. This study emphasizes a need for long‐term studies assessing the clinical effects of systemic absorption. In addition, introduction of alternative filters and formulations that minimize systemic absorption can help mitigate this need.

### 5.2 Terephthalylidene Dicamphor Sulfonic Acid and Drometrizole Trisiloxane

Terephthalylidene dicamphor sulfonic acid (TDSA) and Drometrizole trisiloxane (DT) are additional sunscreen filters safely being used in other countries. Both are organic broad spectrum UV filters patented by L'Oréal and used exclusively in their products [42].

TDSA has a peak UV absorption at 345 nm, whereas DT has two peaks at 303 and 344 nm [42, 43]. TDSA is the water‐soluble form of the filter, whereas DT is the oil‐soluble form. In a study published in 2004, the effects of UV radiation on skin pigmentation were observed in a trial to assess the efficacy of both DT and TDSA, it was found that the two filters show a synergistic effect in preventing skin pigmentation [42]. One study suggests that TDSA can prevent certain aspects of photoaging including hyperpigmentation, pyrimidine dimer formation, p53 protein accumulation, delayed type hypersensitivity reactions, isomerization of urocanic acid, alteration of Langerhans cells, and polymorphous light eruption [44].

Like bisoctrizole and bemotrizinol, TDSA is also known to have little absorption into the dermis, according to a clinical trial published in 2003. The mean in vitro absorption over 24 h, considering the amount found in the dermis and the receptor fluid was 0.16% of the applied dose. In vivo pharmacokinetic results concluded that < 0.1% of TDSA was absorbed systemically [45]. Although it has been tested in vitro, it has not undergone MUsT GRASE requirements for FDA consideration.

This low systemic absorption rate highlights TDSA's safety profile, decreasing the potential for systemic side effects compared to other filters with higher absorption rates. In addition, since TDSA remains primarily on the surface of the skin, it is likely to maintain a higher degree of efficacy as a UV blocker.

## 6 Discussion

Skin cancer remains one of the most common types of malignancies in the United States. It is well‐known that sun exposure plays a significant role in the development of skin cancers, including melanoma. Beyond cancer, exposure to ultraviolet (UV) radiation also accelerates skin aging, contributing to wrinkles and hyperpigmentation. The use of effective sunscreens is one of the most accessible and powerful tools for preventing skin cancer and mitigating photoaging.

However, the current regulatory landscape for sunscreen filters in the U.S. is outdated and does not allow for the use of New Approach Methods for toxicology testing or ISO standards. This leads to limited options for consumers seeking optimal protection against UV radiation. The process to approve new filters can take years and has potentially affected the overall health of Americans. Countries with more streamlined approval processes have access to a broader array of advanced filters that provide superior protection, particularly against UVA radiation. This review highlights the importance of modernizing U.S. sunscreen regulations to incorporate advanced filters used in other countries, which may help reduce both skin cancer rates and visible signs of aging.

In addition, there are health equity concerns related to the access of safe and effective sunscreen products. The stringent FDA approval process limits the availability of advanced sunscreen filters, particularly for low‐income populations who may not have the means to import foreign products. Also, certain populations—especially those with darker skin tones—are often underserved by available U.S. mineral sunscreens, which can leave visible white casts on the skin, further discouraging use. The global availability of more effective and aesthetically pleasing sunscreens from the EU and Asia may exacerbate these disparities unless U.S. regulations are updated to allow for a wider range of effective products.

The reliance on older filters, many of which fail to meet their labeled SPF and UVA protection claims, compromises public trust in sunscreen products. Consumers may be unaware that the products they rely on for sun protection are not as effective as those available internationally. This erosion of trust can lead to reduced usage of sunscreens, increasing the risk of skin damage and skin cancer.

There is evidence to suggest that the sunscreen filters used in the EU and countries like Korea offer more comprehensive UV protection compared to those approved in the U.S. For instance, Korean sunscreens often utilize filters such as bemotrizinol, bisoctrizole, DT, and TDSA, which provide superior UVA and UVB protection compared to U.S. filters like oxybenzone and octinoxate. This fact, plus the studies showing that U.S. sunscreens fail to meet their labeled SPF and UVA protection factor claims, further emphasizes the need for regulatory updates.

## 7 Recommendations

To ensure that U.S. consumers have access to the best possible sun protection, it is imperative that the FDA modernizes its sunscreen regulations. This could include approving newer filters already in use abroad, such as bemotrizinol, bisoctrizole, TDSA, or DT, which have been shown to provide enhanced protection with minimal systemic absorption.

Additionally, the FDA could adopt the PA system, which would offer consumers more information about a product's UVA protection capabilities. This system makes it easier for consumers to understand the level of protection offered against UVA rays. Adopting this system would help bridge the knowledge gap and empower consumers to make more informed choices about their sun protection. Streamlining the approval process for sunscreens to balance safety and efficacy while addressing health disparities should be a priority to reduce the growing incidence of skin cancer and mitigate photoaging in the general population.

The FDA should also consider streamlining its approval processes to balance rigorous safety standards with timely access to advanced sunscreens. This could involve adopting a more flexible approach to safety assessments, like the cosmetic regulations in Europe and Asia, without compromising consumer safety. The FDA could also adopt the ISO sunscreen efficacy standards, specifically ISO 23675:2024 and ISO 23698:2024, which introduce innovative methods that could enhance the accuracy and ethical considerations of sunscreen testing [46, 47] (23698). By reducing the time and complexity of the approval process, the FDA can encourage innovation and ensure that the latest UV protection technologies are available to the public sooner. This may include simplifying the data requirements for filters with a long history of safe use internationally or introducing a fast‐track approval pathway for promising new filters.

## 8 Conclusion

Modernizing U.S. sunscreen regulations is essential for addressing the increasing incidence of skin cancer and improving public health outcomes. By adopting international best practices and incorporating advanced UV filters already approved in Europe and Asia, the FDA can enhance the effectiveness and availability of sunscreens in the United States. This regulatory update would ensure that American consumers have access to the most advanced and reliable UV protection, reducing their risk of UV‐related skin damage and skin cancer.

The adoption of globally recognized filters and the alignment of U.S. regulatory practices with those of Europe and Asia would significantly benefit public health. Providing consumers with better sunscreen options would lead to improved UV protection, lower rates of photoaging, and a reduction in the incidence of skin cancer. Moreover, modernizing the regulatory framework would restore public trust in sunscreen products, ensuring that they deliver the protection they promise.

Reforming the FDA's approach to sunscreen regulation is a critical step toward enhancing public health and reducing the burden of skin cancer. By learning from the regulatory successes of Europe and Asia, the U.S. can provide its citizens with safer, more effective, and aesthetically pleasing sunscreen options, marking a significant advancement in public health and skin cancer prevention.

In conclusion, this review underscores a critical need to reevaluate and update U.S. sunscreen regulations. A reevaluation of the U.S. sunscreen approval process balancing safety, efficacy, and public health outcomes is recommended. Incorporating more advanced filters and broadening access to safe, effective, and cosmetically appealing sunscreens will not only reduce skin cancer incidence but also improve overall skin health.

## Author Contributions

C.W.T. conceived the topic, conducted the literature review, and drafted the manuscript. L.T. provided feedback and approved the final version of the manuscript.

## Ethics Statement

The authors have nothing to report.

## Conflicts of Interest

The authors declare no conflicts of interest.

## Data Availability

The authors have nothing to report.
